# Airway reconstruction in children

**DOI:** 10.4103/0971-9261.57699

**Published:** 2009

**Authors:** Sanjay R. Rao, Ashley D'Cruz, Vinay Jadhav

**Affiliations:** Department of Pediatric Surgery, Narayana Hrudayalaya, Bangalore, India

**Keywords:** Airway, reconstruction, subglottic stenosis, tracheostomy

## Abstract

**Aim/Background:**

Airway anomalies are infrequent but potentially life threatening in children. A program to care for these difficult children was set up at our institution, and this paper summarizes our experience.

**Methods:**

A total of 34 children were enrolled in the program over a period of three years. These children were evaluated as per the standard protocols. Treatment was individualized.

**Results:**

Of these 34 children, 28 had their airways restored and are doing well. Four children continue to remain on tracheostomy and two will require long term tracheostomy. There were two deaths. All children are under surveillance as there is a risk of recurrence.

**Conclusions:**

Airway anomalies are complex problems with significant morbidity and mortality. Current therapeutic modalities allow for good results. Most children were successfully decannulated and did well.

## INTRODUCTION

Airway anomalies in children are potentially life threatening. Common causes are congenital (tracheal stenosis) and acquired (subglottic stenosis post intubation). Children could present acutely (with stridor and acute respiratory insufficiency) or chronically (recurrent airway spasms, lower respiratory tract infection (LRTI), noisy breathing) at any time during childhood. Emergency management includes airway access - either intubation or tracheostomy.

Subsequently, evaluation is followed by definitive therapy to restore the airway and decannulate the child. An airway anomalies service has been setup in our institute to manage these children in a comprehensive manner. This article summarizes our early experiences.

## MATERIAL AND METHODS

This is a study of observations on a cohort of patients referred to the airway team at our hospital. The data was collated in a prospective manner in a custom database which included demographics, presenting symptoms, physical findings, investigations, surgical details and outcome. Data was analyzed and results have been presented in this study.

These children were evaluated using a standard data sheet. All children had a detailed history taken; emphasis was on the nature of airway obstruction symptoms, previous intubations and airway interventions, features of gastro esophageal reflux and current airway status.

All children underwent a micro laryngo-bronchoscopy under general anesthesia; initially with spontaneous respiration and later paralysis if required. Children with airway foreign bodies and purely inflammatory pathology were excluded. A detailed assessment of the anatomy of the entire airway was made and cultures were collected as per the clinical indications. Based on this preliminary assessment, select patients underwent further imaging with computerized tomography (CT) scans and ECHO cardiograms. This was usually indicated with extrinsic compression of the airway was suspected. Gastro esophageal reflux studies were done when clinically indicated.

Therapy was individualized; degree of symptoms, nature and degree of airway obstruction and nature of previous airway interventions were primary factors considered. All symptomatic and/or higher grades were offered surgery. Minor degrees of obstruction that were asymptomatic were treated conservatively. Prerequisites for surgery were absence of - active respiratory tract infection and pathogenic bacteria in throat swabs/tracheal secretions; good control of gastro esophageal reflux. In children who had undergone cardiac surgery, the cardiac status should be stable and the child not oxygen-dependant.

## RESULTS

Thirty four patients with airways anomalies were evaluated. Girls accounted for 50% of cases. The age ranged from newborn to 16 years, the median age being 2 ½ years. The pathological lesions are listed in Table 1. Various interventions performed are listed in Table 2.

**Table 1:** Etiologies of Airway Obstruction

Diagnosis	Number
Subglottic stenosis	13
Tracheal Stenosis	4
Laryngomalacia	5
Tracheobroncomalacia	8
Vascular compression	4
Total	34

**Table 2:** Treatment Modalities

Diagnosis	Total	Surgery	Conservative	Type of surgery
Subglottic Stenosis	13	10	3	8 Laryngo-tracheal reconstruction (LTR)
				2 Tracheotomy awaiting LTR
Tracheal stenosis	4	3	1	2 Tracheal resection
				1 Slide tracheoplasty
Laryngomalacia	5	4	1	4 tracheostomy of whom 3 decanulated and 1 awaiting supraglottoplasty
Tracheal-bronchomalcia	8	2	6	1 tracheal T tube stenting
				1 tracheostomy
Vascular compression	4	3	1	1 aortic arch division
				2Main Pulmonary Artery(MPA), Left Pulmonary Artery (LPA) plication

Fourteen children had tracheostomy, 10 have been decannulated. Of the four continuing to have tracheostomy, two are awaiting definitive repairs and decannulation, and the other two are candidates for long term tracheostomy. Both these children have extensive tracheomalacia. They have had complex cardiac surgery with very stormy postoperative periods. Two children had tracheal T tubes. In one of these, a two-year-old boy, tracheal T tube was converted to a tracheostomy due to recurrent life threatening T tube blocks. The other child, a five-year-old, continues to be on the T tube.

There were two deaths in the series. One was the boy who had slide tracheoplasty for complete tracheal rings. This child also had a tetrology of Fallot. He underwent slide tracheoplasty and Blalock-Taussig (BT) shunt under cardiopulmonary bypass. The BT shunt required re-exploration for bleeding. On the eighth day post-op, he developed a thrombosis of the BT shunt and developed a pulmonary hypertensive crisis and succumbed. The airway repair itself was intact. The other child who succumbed had a severe segmental malacia of the left main bronchus and was planned for stenting. She also had a complex ventricular septal defect (VSD) requiring repair. However, the parents refused consent for the procedure. The child remained on prolonged ventilation and succumbed to infectious complications.

Follow-up ranged from six weeks to three years with a median follow up of 12 months.

All those whose treatment is completed had competent airways and good voice. They continue to remain on surveillance.

## DISCUSSION

Increasing use of ventilation and other aggressive interventions resulted in a group of children with airway morbidity. This morbidity is potentially life threatening and has a serious effect on the quality of the child's life. A comprehensive effort is required to limit these problems and treat children who have developed them. Any child who develops airway symptoms and asthma after a period of intubation must be evaluated for airway obstructions. Evaluation attempts to define the exact anatomy of the airway, look for and treat co-morbid conditions such as gastro esophageal reflux. Specific airway intervention is individualized. A variety of treatment options are available. These include tracheostomy, T tube placements, laryngo-tracheal expansion and resection and tracheal expansions and resections.

Tracheostomy is a life saving intervention and allows secure access to airway. Tracheostomy is also performed in chronically ventilated patients to facilitate weaning. In the absence of an associated airway anomaly, most children are decannulated in a few weeks. Inability to decannulate indicates the need for a formal airway evaluation. Domiciliary care of tracheostomy is possible even in rural areas. Motivation of the family is the biggest determinant of efficient domiciliary tracheostomy care.[1] However, complications are frequent and an experienced team is essential to care for children with tracheostomy.

### Subglottic Stenosis (SGS):

**Figure 1:**
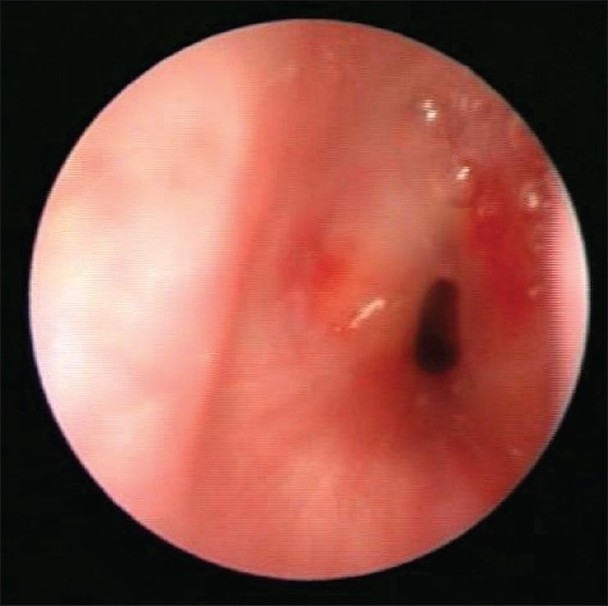
Post-intubation Subglottic Stenosis

The subglottic area is the narrowest part of the airway. Endotracheal intubation results in the mucosa and sub mucosa of the subglottic area getting compressed and ischemic between the tube and the complete cricoid ring. This ischemia results in ulceration and scarring of this area. This is the commonest cause of SGS in children [Figure 1]. Incidence of SGS after cardiac surgery is 2.3%.[2] Congenital SGS is also well known and is thought to be due to an improper canalization of this region during organogenesis.

**Figure 2:**
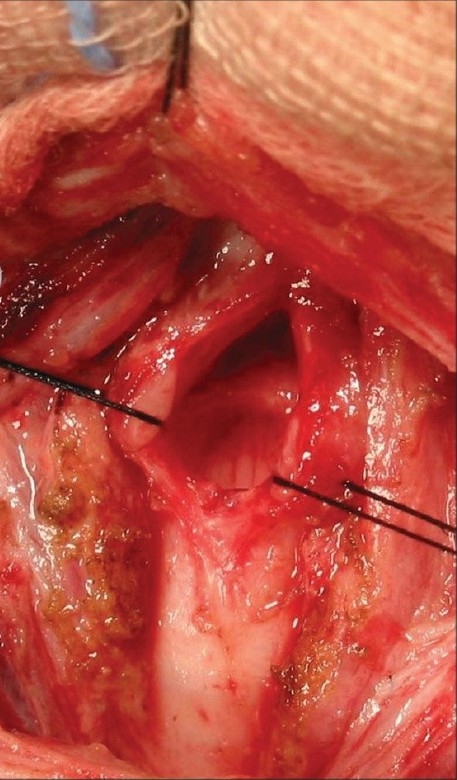
Surgery showing cricoid and subglottic ring split open. (this gap is held open with a costal cartilage graft)

SGS is graded as per the Cotton Myer scale[3] (grade 1 being 0-50% narrowing to grade 4 being total occlusion). Milder degrees of SGS can be managed conservatively. However, major degrees of stenosis and those that are symptomatic need surgery. These surgeries could be expansion operations (anterior cricoid split with or without cartilage grafts) [Figure 2] or resection operations (cricotracheal resections).[4] Decannulation rates of above 90% are achieved overall, even in patients who have had multiple previous interventions. The congenital stenosis does better as there is less scarring.

### Congenital Tracheal Stenosis (CTS):

This is an infrequent anomaly. There was one child with this problem. He was from Nigeria and had an associated tetrology of Fallot. He was significantly symptomatic and the tracheal stenosis was about 50% of his total tracheal length and was more than 60% of the natural tracheal lumen. He underwent slide tracheoplasty under cardiopulmonary bypass and a BT shunt was performed at the same time. Unfortunately, the BT shunt needed to be re-explored for a bleed on the first post-op day. On the eighth post-op day he succumbed to a spell secondary to a blocked BT shunt. The airway repair continued to remain intact. Surgical repair remains the traditional means of treating this anomaly.[5] However, Wei *et al*. reported a group of six children managed conservatively. They concluded that a select group of children who have a short segment stenosis and a lumen not lesser than 60% of the normal tracheal diameter could be managed conservatively.[6]

### Acquired Tracheal Stenosis:

Tracheal injuries from intubation/instrumentation account for over 90% of all tracheal stenosis. Of the three cases of tracheal stenosis, in this series, two were a consequence of previous intubations. The two children, with post-intubation tracheal stenosis, had previous interventions - one had dilatations and the other, who was trachestomized, had laser ablation. In both cases, resection of the fibrotic segment was required and resolved the matter. Grillo[7] reported successful repair in 17 of 20 children with tracheal stenosis. Other modalities used in this group included use of tracheostomy tubes and T tubes.

### Tracheo Broncomalacia (TBM):

The cartilage in major airways provides it with a degree of rigidity that allows laminar airflow during respiration. Weakness of this cartilage frame work increases compliance of major airways. TBM may be primary but, more often, is secondary to other congenital anomalies (esophageal atresia, cardiac anomalies etc). Prolonged ventilation and multiple airway interventions too can cause TBM. It is a significant cause for failure of extubation in the postoperative period in children after cardiac surgery, in most, the condition is self limiting. However, intervention is required in the presence of life threatening airway compromise and failure to decannulate. These interventions include internal stenting (with tracheostomy tubes, T tubes, stents), aortopexy and procedures to remove vascular structures that are compressing the airways.[8]

In summary, these children have complex and often multiple problems. Careful evaluation of the entire airway is required in tailor treatment. With an individualized and comprehensive approach to treatment, most children can successfully have their normal airways restored.
